# Qualitative/Chemical Analyses of Ankaferd Hemostat and Its Antioxidant Content in Synthetic Gastric Fluids

**DOI:** 10.1155/2016/8957820

**Published:** 2016-01-26

**Authors:** Ahmet Koluman, Nejat Akar, Umit Y. Malkan, Ibrahim C. Haznedaroglu

**Affiliations:** ^1^National Food Reference Laboratory, Department of Mineral Analyses, Ministry of Food Agriculture and Livestock, 06170 Ankara, Turkey; ^2^Department of Pediatric Hematology, TOBB-ETU Hospital, 06570 Ankara, Turkey; ^3^Department of Adult Hematology, Hacettepe University Medical School, 06100 Ankara, Turkey

## Abstract

*Introduction.* Ankaferd hemostat (ABS) is the first topical haemostatic agent involving the red blood cell-fibrinogen interactions. The antihemorrhagic efficacy of ABS has been tested in controlled clinical trials. The drug induces the formation of an encapsulated complex protein web with vital erythroid aggregation. The aim of this study is to detect the essential toxicity profile and the antioxidant molecules inside ABS.* Methods.* The pesticides were analyzed by GC-MS and LC-MS. The determination by ICP-MS after pressure digestion was performed for the heavy metals. HPLC was used for the detection of mycotoxins. Dioxin Response Chemically Activated Luciferase Gene Expression method was used for the dioxin evaluation. TOF-MS and spectra data were evaluated to detect the antioxidants and other molecules.* Results.* TOF-MS spectra revealed the presence of several antioxidant molecules (including tocotrienols, vitamin E, tryptophan, estriol, galangin, apigenin, oenin, 3,4-divanillyltetrahydrofuran, TBHQ, thymol, BHA, BHT, lycopene, glycyrrhetinic acid, and tomatine), which may have clinical implications in the pharmacobiological actions of ABS.* Conclusion.* The safety of ABS regarding the presence of heavy metals, pesticides, mycotoxins, GMO and dioxins, and PCBs was demonstrated. Thus the present toxicological results indicated the safety of ABS. The antioxidant content of ABS should be investigated in future studies.

## 1. Introduction

Ankaferd hemostat, ABS, is the first topical haemostatic agent involving the red blood cell- (RBC-) fibrinogen interactions. ABS comprises a standardized mixture of the plants* Thymus vulgaris*,* Glycyrrhiza glabra*,* Vitis vinifera*,* Alpinia officinarum*, and* Urtica dioica* (reviewed in [1]). The overall hemostatic effects of ABS depend upon the protein agglutination and polymerization modulating the erythroid aggregation within the vascular endothelial system [1, 2]. Prohemostatic and antithrombin activities of Ankaferd hemostat are linked to the fibrinogen gamma chain and prothrombin [2]. ABS induces the formation of an encapsulated complex protein web with vital erythroid aggregation covering the entire physiological hemostatic process [3]. The unique hemostatic properties of ABS provide a balanced hemostasis representing a basis for physiological wound healing [4–8]. The structural and functional properties of the proteins related to the biological effects of ABS have previously been investigated with the functional proteomics [9] and transcriptomics [10] analyses.

Randomized clinical trials (RCT) indicated the safety and efficacy of ABS for the topical control of clinical hemorrhages in a wide variety of settings [11–18]. Likewise, the cumulating preliminary data points out the expanding spectrum of ABS. For instance, a recent clinical study by Patiroglu et al. has demonstrated that oral topical ABS use at the beginning of chemotherapy could provide less oral mucositis when compared to the control group in the pediatric patients with cancer [19]. Moreover, experimental antineoplastic activities of ABS have been shown in rats and cancer cell lines [20–22].

ABS may be used as a supportive agent together with the antituberculosis treatments during debridement of multiple drug-resistant* M. tuberculosis *caused osteomyelitis and lymphadenitis lesions [23]. Oral/endoscopic ABS administration has already been performed in the gastrointestinal system (GIS) hemorrhages [24–27]. Moreover, ABS is active against multiresistant bacteria, such as methicillin-resistant* Staphylococcus aureus *(MRSA),* Enterococcus *spp., generic* Escherichia coli*,* Klebsiella *spp.,* Acinetobacter *spp., and* Pseudomonas *spp., as well as fungi such as* Aspergillus *spp.,* Mucor *spp., and* Candida albicans* [28–31]. Therefore, anti-infective properties of the ABS may be considered for the supportive usage of this agent in numerous infectious disorders in the future.

Expanding clinical spectrum of ABS is evident. Thus, additional toxicological data for the possible topical and/or systemic usage of this unique haemostatic agent are needed. The aim of this study is to detect essential toxicity profile (heavy metals, pesticides, mycotoxins, GMO (genetically modified organisms), dioxins, and polychlorinated biphenyls (PCBs) analyses). Furthermore, the antioxidant molecules inside ABS are also searched. The antioxidant system might be related to the ABS-induced pleiotropic actions (hemostasis, infection, cellular proliferation, and vessel wall dynamics) [1, 32, 33]. Likewise, ABS attenuated the oxidative and inflammatory changes caused by ASA-induced gastric mucosal damage in a previous study [32]. Thus, we also aimed to investigate the fate of antioxidant content of ABS when exposed to the synthetic gastric fluid.

## 2. Materials and Methods

Ten milliliters (mL) of ABS sample was transferred to the laboratory (Lab) under cold chain in a residue-free sterile tube. The sample was taken into analyses within 30 minutes after the handling of ABS in the Lab.

*For the analyses of heavy metals,* the organic molecules in 0.3 mL of ABS were digested using NHO_3_ and H_2_O_2_ under pressured microwave oven and samples were read in ICP-MS according to the method referred by the Nordic Committee on Food Analysis (NMKL) 186 (NMKL trace elements—As, Cd, Hg, Pb, and other elements. The specification by ICP-MS after pressure digestion was conducted (NMKL 186) (2007)).

*For the pesticides analyses,* 1 mL of ABS sample was taken for extraction of pesticides. After the extraction, pesticides were analyzed in Gas Chromatography Mass Spectroscopy (GC-MS) and Liquid Chromatography Mass Spectroscopy (LC-MS) using the methods related with the analytes (EPA 1668.821-R-00-002, December 1999).

*For the mycotoxins detection,* 1 mL of ABS sample was used for the extraction and the extracts were injected to High Performance Liquid Chromatography (HPLC) using the method of AOAC (AOAC Official Method 999.07).

*For the GMO (genetically modified organisms) analyses, *1 mL of ABS sample was used to extract DNA with Qiagen DNeasy Blood & Tissue Kit and QIAprep Spin Miniprep Kit. After extraction gel electrophoresis was held using the method International Standardization Organization, ISO 24276 (ISO 24276: Foodstuffs—Methods of analysis for the detection of genetically modified organisms and derived products, 2009) and ISO 21569 (ISO21569: Foodstuffs—Methods of Analysis for the Detection of Genetically Modified Organisms and Derived Products—Qualitative Nucleic Acid Based Methods, 2005).

*Regarding the dioxin analyses, *3 mL of ABS sample was used for extraction and Dioxin Response Chemically Activated Luciferase Gene Expression (DR Calux) method was used for the dioxin evaluation.

*For the PCB analyses, *EPA 1668 and CEN 1528 methods have been utilized for the evaluation.

*In order to detect the antioxidants and other molecules*, 1 mL of ABS sample was diluted and given to Time of Flight Mass Spectroscopy and spectra were evaluated using the online library. The antioxidant molecules were evaluated using the method determined by AOAC 983.15, 2007.

For the detection of* fate of ABS in synthetic gastric fluid (SGF)*, synthetic gastric fluid was prepared as previously described by Molly et al. [34]. SGF exposure tests were carried out after the inoculation of ABS on the minutes of 1, 5, 10, and 15. Triplicate samples of 2 mL of ABS were mixed with 10 mL of SGF (pH adjusted to 1.0 with HCl) and each was used in the tests. The buffering effect of the ABS was controlled; the pH value of the mix was adjusted to 1.0 as needed.

*Apigenin detection* was held according to the method published by Xiao et al. [35]. Glass-stoppered test tubes (15 cm^3^) with a Teflon-coated magnetic stirrer were used to make saturated solutions (about 8.0 cm^3^) of apigenin or apigenin 7-O-rhamnosylglucoside with excess solid solute (about 0.3 g) in these solvents. The test tubes were closed up with paraffin to avoid evaporation of solvents. After that, the test tubes were put straight in a constant-temperature thermostatic bath (PolyScience Circulators 9501, USA) with a temperature accuracy of ±0.05 K and stability of 0.01 K. Undissolved solid and solution were permitted to settle about 24 to 36 h to ensure equilibrium before sampling. For each test tube, three samples of approximately 0.2 to 0.3 cm^3^ were withdrawn from the clear saturated solution using preheated glass syringes.

The glass syringe with saturated solution was measured by a Sartorius CP225D analytical balance with an accuracy ±0.01 mg. In order to prevent evaporation of solvents during the weighing process, the needle was locked with silicone rubber. The saturated solution was added to the volumetric flask (10 cm^3^) immediately to avoid precipitating. Afterward, the mass of glass syringes with the remaining solution was measured. The mass of saturated solutions that were placed into volumetric flasks can be found. The solutions of samples used to examine were diluted to mark with methanol. The reference standard solution containing 16 *μ*g·cm^−3^ of apigenin and 15 *μ*g·cm^−3^ of apigenin 7-O-rhamnosylglucoside was set in methanol, respectively. To determine the concentration of apigenin and apigenin 7-O-rhamnosylglucoside, an HPLC system (LC-10AD, Shimadzu, Japan) was used. All chromatographic studies were done on Diamonsil ODS C18 column (250 mm × 4.6 nm, 5 *μ*m) with the wavelength of detector set at 256 and 333 nm, respectively. The mobile phase was composed of acetonitrile and an aqueous solution having a volume fraction of 0.1% of phosphoric acid in a volume ratio of 35 : 65 and 25 : 75 at a flow rate of 1.0 cm^3^ · min^−1^. The injected volumes of sample and reference standard solutions were 0.020 cm^3^. All chromatograph procedures were done at room temperature.

*Tocopherol and tocotrienol detection* was done according to the method described by Albahrania and coworkers [36]. All samples were organized as follows: 100 *μ*L of sample and 100 *μ*L of Milli-Q water were added to a borosilicate glass tube and vortexed. Next, 200 *μ*L of methanol having the deuterated internal standard was injected to this combination, vortexed, and then equilibrated for 10 min at room temperature. Analytes were extracted with addition of 1 mL of HPLC grade hexane to the combination and then vortexed extensively before centrifugation at 3000 rpm for 5 min at room temperature. The organic layer was moved to a new glass tube and dried below a stream of nitrogen gas at room temperature and then reconstituted in 250 *μ*L methanol. This sample preparation process was performed under depressed light. The reconstituted sample (1 *μ*L) was added to the Agilent LC-MSMS system (Agilent 1290 Infinity LC and Agilent 6490 Triple Quadrupole Mass Spectrometer, Agilent Technologies, Victoria, Australia). Chromatographic isolation of analytes was conducted with a Pursuit^*®*^  XRs C18 column (20 mm × 2 mm × 3 *μ*m) and MetaGuard 2.0 mm Pursuit XRs 3 *μ*m C18 from (Agilent Technologies). The mobile phases consisted of mobile phase A (MP-A), 0.1% formic acid in Milli-Q water containing 2% methanol and mobile phase B (MP-B), 0.1% formic acid in methanol. A constant flow rate of 0.2 mL/min and a gradient profile from 80% to 100% of MP-B were employed.

## 3. Results

*The quantitative analyses of the heavy metals (Pb, Cd, Hg, As, and Bor) in the ABS sample* revealed that ABS does not contain Pb, Cd, Hg, and As (the concentrations of the heavy metals were Pb: 0,008 ppb; Cd: 0,000 ppb; Hg: 0,004 ppb; As: 0,000 ppb, and Bor: 0,000 ppb, resp.).

*The chromatographic analyses of the pesticides analyses in the ABS sample* revealed that ABS does not contain pesticides. The searched specific pesticides (which are not present in ABS) that were analyzed by GC-MS and LC-MS methods were depicted in Table 1.

*The analyses focusing on mycotoxins detection in the ABS sample* via using the HPLC method revealed that ABS does not contain mycotoxins (namely, Aflatoxin B1, Aflatoxin B2, Aflatoxin G1, and Aflatoxin G2; total aflatoxins were not present in ABS).

*The GMO analyses regarding the detection of genetically modified organisms in the ABS sample* indicated that any plasmid extraction had not been realized, indicating that ABS does not have a GMO process during the preparation process.

*The dioxin analyses in the ABS sample* revealed that ABS does not contain toxic dioxin and dioxin-like chemical compounds.

**Figure 1:**
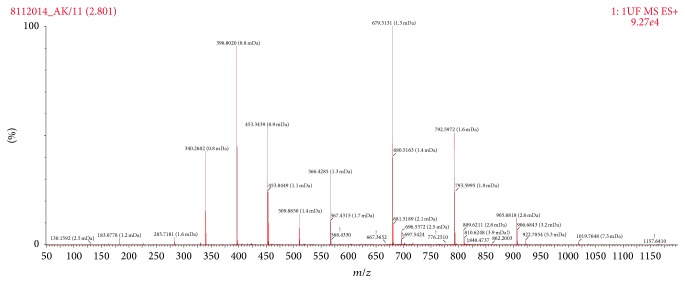
Antioxidant content of Ankaferd hemostat (ABS) detected by Time of Flight Mass Spectroscopy (TOF-MS). The complicating background noise due to the complex molecular library of ABS prevented the exact specifications of the molecules evaluated from the TOF-MS. Nevertheless, careful analyses on TOF-MS spectra exactly revealed the presence of some antioxidants such as tocotrienols, members of the vitamin E family, tryptophan, estriol, galangin, apigenin, oenin, 3,4-divanillyltetrahydrofuran, tertiary butylhydroquinone (TBHQ), thymol, BHA (butylated hydroxyanisole), BHT (butylated hydroxytoluene), lycopene, enoxolone/glycyrrhetinic acid or glycyrrhetic acid, and tomatine.

The complicating background noise due to the complex molecular library of ABS prevented the exact quantitative specifications evaluated from the Time of Flight Mass Spectroscopy (TOF-MS) and spectra, regarding the presence of* the antioxidants and other specific molecules in the ABS sample*. Nevertheless, careful analyses on TOF-MS spectra exactly revealed the presence of some antioxidants (such as tocotrienols, members of the vitamin E family, tryptophan, estriol, galangin, apigenin, oenin, 3,4-divanillyltetrahydrofuran, tertiary butylhydroquinone (TBHQ), thymol, BHA (butylated hydroxyanisole), BHT (butylated hydroxytoluene), lycopene, enoxolone/glycyrrhetinic acid or glycyrrhetic acid, and tomatine) (Figure 1). The concentrations of the antioxidants and other specific molecules in the ABS sample have not been affected after the exposure to the synthetic gastric fluid.

## 4. Discussion

In this study, the safety profile for ABS focusing on the presence of heavy metals, pesticides, mycotoxins, GMO (genetically modified organisms), and dioxin has been demonstrated. ABS is composed of standardized plant extracts including* Alpinia officinarum*,* Glycyrrhiza glabra*,* Thymus vulgaris*,* Urtica dioica*, and* Vitis vinifera*. ABS-induced pharmacological modulation of essential erythroid proteins (ankyrin, spectrin, and actin) can cause vital erythroid aggregation via acting on fibrinogen gamma. ABS also has pleiotropic effects particularly in the tissue healing and has anti-infective properties [1, 3, 9, 10, 33, 37, 38]. The toxicological results of the present study have provided the basis for the future ABS clinical trials in the fields of clinical hemostasis, wound healing, burn treatment, and anti-infective and antineoplastic approaches [4, 22, 39–47]. Moreover, next-generation RBC-related hemostatics, such as ABS nanohemostat, have already been designated in the essential treatment of life-threatening bleedings by restoring physiological hemostasis via acting on RBCs [48, 49]. The safety profile for ABS obtained from this present report opens new avenues for the nanomedicinal production of novel chimeric plant-based hemostatic agents as of ABS nanohemostat [48, 49]. Although our toxicological study should be accepted as a clue for the safety of the plant-based topical hemostatics, the issue is far from being completely resolved. For the active substances of plant extracts, exact safe levels of potential human exposure cannot be determined due to the GRAS (*generally regarded as safe*) ingredients (*FDA, GRAS List of Some Chemicals and Plants *(http://www.accessdata.fda.gov/scripts/cdrh/cfdocs/cfcfr/CFRSearch.cfm?fr=182.20)).

In our present study, TOF-MS spectra revealed the presence of several antioxidant molecules (including tocotrienols, members of the vitamin E family, tryptophan, estriol, galangin, apigenin, oenin, 3,4-divanillyltetrahydrofuran, tertiary butylhydroquinone (TBHQ), thymol, BHA (butylated hydroxyanisole), BHT (butylated hydroxytoluene), lycopene, enoxolone/glycyrrhetinic acid or glycyrrhetic acid, and tomatine), which may have clinical implications in the pharmacobiological actions of ABS hemostatic agent. Furthermore, the concentrations of the antioxidants and other specific molecules in the ABS have not been affected after the exposure to the synthetic gastric fluid. Those findings have provided the basis for the future ABS clinical trials that will be designed for the oral systemic administration to control hemorrhages of the GIS tract. The contact of the gastric mucosa with the destructive factors could produce pathological changes such as ongoing inflammatory process, hemorrhagic erosions, and even acute ulcers. Hasgul and coworkers investigated the contribution of reactive oxygen species (ROS) in acute gastric mucosal injury by acetylsalicylic acid (ASA) and the effects of ABS in a rat model [32]. They demonstrated the biological effects of ROS as assessed by determining the tissue and plasma levels of malondialdehyde, the products of lipid peroxidation, as well as the activity of superoxide dismutase and the scavenger of reactive oxygen species produced by ASA in the experiment group. Furthermore, MPO activity as well as the NO and tumor necrosis factor (TNF) levels also demonstrated important improvement by ABS therapy. In their study, ABS attenuated the oxidative and inflammatory changes caused by ASA-induced gastric mucosal injury in rats [32]. Our present findings about the antioxidants of ABS content that had not been negatively affected after the exposure to the acidic gastric fluid further enlightened those previous experiments. The topical effects of ABS on GI system such as esophagus, stomach, intestines, liver, spleen, and vessels have been extensively searched in previous animal models [32, 40, 41, 50–58]. All of those data represent the basis for future clinical trials regarding the efficacy and safety of ABS in a wide variety of GI disorders such as infection, inflammation, and cancer.

Experimental trials indicated that ABS is effective in wound healing. Aktaş et al. have demonstrated that application of ABS after the tooth extraction can cause increased secretion of collagen type 1, collagen type 3, smooth muscle actin, fibronectin, beta 2 microglobulin, vascular endothelial growth factor, and cyclooxygenase-2, which are very effective in early phases of wound healing. ABS had provided rapid wound healing with the actions of those molecules [42]. Moreover, ABS can cause leukocyte infiltrations, vascularization, and fibroblast proliferation in the mucosal tissue that could facilitate the wound healing process [4]. Kaya and coworkers topically used ABS on the burning tissue and provided decrements in the wound diameter and inflammation, accelerated tissue fibrosis, and wound contraction [6]. The antioxidant molecules of ABS detected in our study may be placed into those complicated interactions. However, the exact ABS mechanism-of-action in the wound healing process should be investigated in future experimental studies.

Apigenin has been detected in the molecular library of ABS in this present study. Apigenin has inhibitory effects against the snake venom metalloproteases, the main component accountable for the hemorrhage and tissue degradation at the bitten site [59]. Therefore, the already established antihemorrhagic efficacy of ABS and its apigenin content shall be searched in future toxicological experiments. Likewise, tocopherol and tocotrienol have been detected inside ABS in our study. Tocotrienol is an antioxidant which has been found commercial application as a food additive and health supplement. Tocotrienol 2% in powdered basal diet in rats had led to the hemorrhage of several organs by week 50 in a previous study [60]. Tocotrienol can induce apoptosis and suppress cellular proliferation [61–64] and has antiangiogenic effects [65]. Therefore, tocotrienol is considered as a potential anticancer agent [66]. Similarly, ABS can induce apoptosis, regulates cellular proliferation [21, 67–69], and decreases tumor vascularization [52]. Experimental antineoplastic activities of ABS have been shown in rats and cancer cell lines [20–22]. The interrelationships between ABS, its antioxidant content, and tocotrienol should be investigated in future experimental neoplastic studies.

When addressing the safety of a given product, the moieties found inside shall be assessed to determine if the levels are safe for the exposed patients in order to perform a risk assessment, based on the available data. This is a limitation of our present study. Previously, the clinical phase I double-blinded, randomized, cross-over, placebo-controlled clinical study with a 5 days' washout period between the cross-over periods in healthy volunteers indicated the safety of ABS [15]. Controlled clinical trials [11–18] also have not indicated any safety alert in association with the clinical use of Ankaferd hemostat. However, further clinical trials shall be designed to test clinical responses to Ankaferd hemostat as well as the parallel biological tests focusing on the moieties found inside the hemostatic agent to determine if the levels are safe for the exposed patients.

Clinical studies indicated that topical ABS is a safe and effective haemostatic agent for many different types of internal and external bleedings that are resistant to treatment with conventional antihemorrhagic methods [11–18]. The ability of ABS to induce formation of a protein network regulating the RBC-fibrinogen interactions not only makes it an effective hemostatic agent but also confers anti-infective, antineoplastic, and healing modulator properties. WHO Guidelines [70] for good clinical practice (GCP) for trials on pharmaceutical products (*World Health Organization, WHO Technical Report Series, number 850, 1995, Annex 3*) clearly indicated that “*all research involving human subjects should be conducted in accordance with the ethical principles contained in the current version of the Declaration of Helsinki. Three basic ethical principles should be respected, namely justice, respect for persons, and beneficence (maximizing benefits and minimizing harms and wrongs) or non-malfeasance (doing no harm), as defined by the current revision of the International Ethical Guidelines for Biomedical Research Involving Human Subjects or the laws and regulations of the country in which the research is conducted, whichever represents the greater protection for subjects*.” Accordingly, Ankaferd hemostat has been searched in this study for the potential toxic agents regarding the assessment of safety. Present* in vitro* study indicated that no potential toxic substance present in ABS, enabling future* in vivo* studies. The present toxicological results indicating the safety of ABS have provided the basis for the upcoming trials. Future clinical and experimental controlled studies are needed to shed further light on the expanding spectrum of ABS effects in clinical hemostasis, wound healing, burn treatment, and anti-infective and antineoplastic approaches.

## Figures and Tables

**Table 1:** The searched specific pesticides (*which are not present in ABS*) that are analyzed by Gas Chromatography Mass Spectroscopy (GC-MS) and Liquid Chromatography Mass Spectroscopy (LC-MS) methods.

GC-MS	*2-Phenylphenol, Acetochlor, Acrinathrin, Alachlor, Aldrin, Dieldrin, Benfluralin, Bifenox, Bifenthrin, Biphenyl, Bromophos ethyl, Bromophos methyl, Bromopropylate, Captan, Chlordane, Chlorfluazuron, Chlormephos, Chlorothalonil, Chlorpyrifos, Chlorpyrifos-methyl, Cyfluthrin, Cypermethrin, DDT, Deltamethrin, Dichlobenil, Dicloran, Dicofol, Diphenylamine, Endosulfan, Endrin, Fenarimol, Fenchlorphos, Fenitrothion, Fenpropathrin, Fenvalerate, Esfenvalerate, Flucythrinate, Folpet, Fonofos, Formothion, Heptachlor, Hexachlorobenzene, Hexachlorocyclohexane, Indoxacarb, Iprodione, Kresoxim-methyl, Lambda-cyhalothrin, Lindane, Lufenuron, Methoxychlor, Metribuzin, Nitralin, Nitrofen, Nuarimol, Oxyfluorfen, Parathion, Parathion-methyl, Permethrin, Procymidone, Propargite, Prothiofos, Quinomethionat, Quintozene, Spirodiclofen, Tau-fluvalinate, Tecnazene, Tetradifon, Tetrasul, Tolclofos-methyl, Triallate, Trichloronat, Trifloxystrobin, Trifluralin, *and* Vinclozolin*

LC/MS/MS	*Acephate, Acetamiprid, Aldicarb, Ametryn, Amitraz, Atrazine, Azinphos-ethyl, Azinphos-methyl, Azoxystrobin, Benalaxyl, Bensulfuron-methyl, Bitertanol, Boscalid, Bromacil, Bupirimate, Buprofezin, Cadusafos, Carbaryl, Carbendazim, Carbofuran, Carboxin, Carfentrazone-ethyl, Chlorfenvinphos, Chloridazon, Chlorpropham, Chlorsulfuron, Clofentezine, Clothianidin, Coumaphos, Cyanazine, Cyazofamid, Cycloate, Cymoxanil, Cyproconazole, Cyprodinil, Dialifos, Diazinon, Dichlofluanid, Dichlorvos, Diethofencarb, Difenoconazole, Dimethenamid, Dimethoate + omethoate, Dimethomorph, Diniconazole, Diphenamid, Epoxiconazole, Ethiofencarb, Ethiofencarb-sulfone, Ethiofencarb-sulfoxide, Ethion, Ethofumesate, Ethoprophos, Etofenprox, Etrimfos, Famoxadone, Fenamidone, Fenamiphos, Fenazaquin, Fenbuconazole, Fenhexamid, *and* Fenoxycarb Fenpropimorph, Fenpyroximate, Fensulfothion, Fenthion, Flufenoxuron, Fluquinconazole, Fluorochloride, Flusilazole, Flutriafol, Furathiocarb, Haloxyfop, Heptenophos, Hexaconazole, Hexythiazox, Imazalil, Imidacloprid, Iprovalicarb, Isazafos, Isocarbofos, Isofenphos, Iso-malathion, Lenacil, Linuron, Malathion, Mecarbam, Mefenpyr-diethyl, Mephosfolan, Mesosulfuron-methyl, Metalaxyl, Metamitron, Methacrifos, Methamidophos, Methidathion, Methiocarb, Methomyl and Thiodicarb, Methoxyfenozide, Metobromuron, Metolachlor, Metoxuron, Metrafenone, Mevinphos, Molinate, Monocrotophos, Monolinuron, Myclobutanil, Nicosulfuron, Nitenpyram, Ofurace, Oxadixyl, Oxamyl, Oxydemeton-methyl, Paclobutrazol, Penconazole, Pencycuron, Pendimethalin, Phenthoate, Phosalone, Phosfolan, Phosmet, Phosphamidon, Pirimicarb, Pirimiphos-ethyl, Pirimiphos-methyl, Prochloraz, Profenofos, Promecarb, Prometryn, Propamocarb, Propanil, Propaquizafop, Propazine, Propetamphos, Propham, Propiconazole, Propoxur, Propyzamide, Prosulfocarb, Pyraclostrobin, Pyrazophos, Pyridaben, Pyridaphenthion, Pyrimethanil, Pyriproxyfen, Quınalphos, Quizalofop-ethyl, Rimsulfuron, Simazine, Spinosad, Spiroxamine, Sulfotep, Tebuconazole, Tebufenozide, Tebufenpyrad, Tepraloxydim, Terbufos-sulfone, Terbumeton, Terbuthylazine, Terbutryn, Tetrachlorvinphos, Tetraconazole, Thiabendazole, Thiacloprid, Thiamethoxam, Thifensulfuron-methyl, Thiobencarb, Thiophanate-methyl, Tralkoxydim, Triadimefon and Triadimenol, Triasulfuron, Triazophos, Trichlorfon, Tricyclazole, Triflumizole, Triticonazole, Vamidathion, *and* Zoxamide*
